# Supplementary material for: The causal role of circulating inflammatory markers in osteoporosis: a bidirectional Mendelian randomized study
Source: Front Immunol. 2024 Jul 18;15:1412298. doi: 10.3389/fimmu.2024.1412298 (PMC11291241; doi:10.3389/fimmu.2024.1412298)
Supplement: Supplementary file 1 [file DataSheet_1.zip › supplementary material/CIM-OSTEOPOROSIS /GCST90274822.csv_forest.pdf]

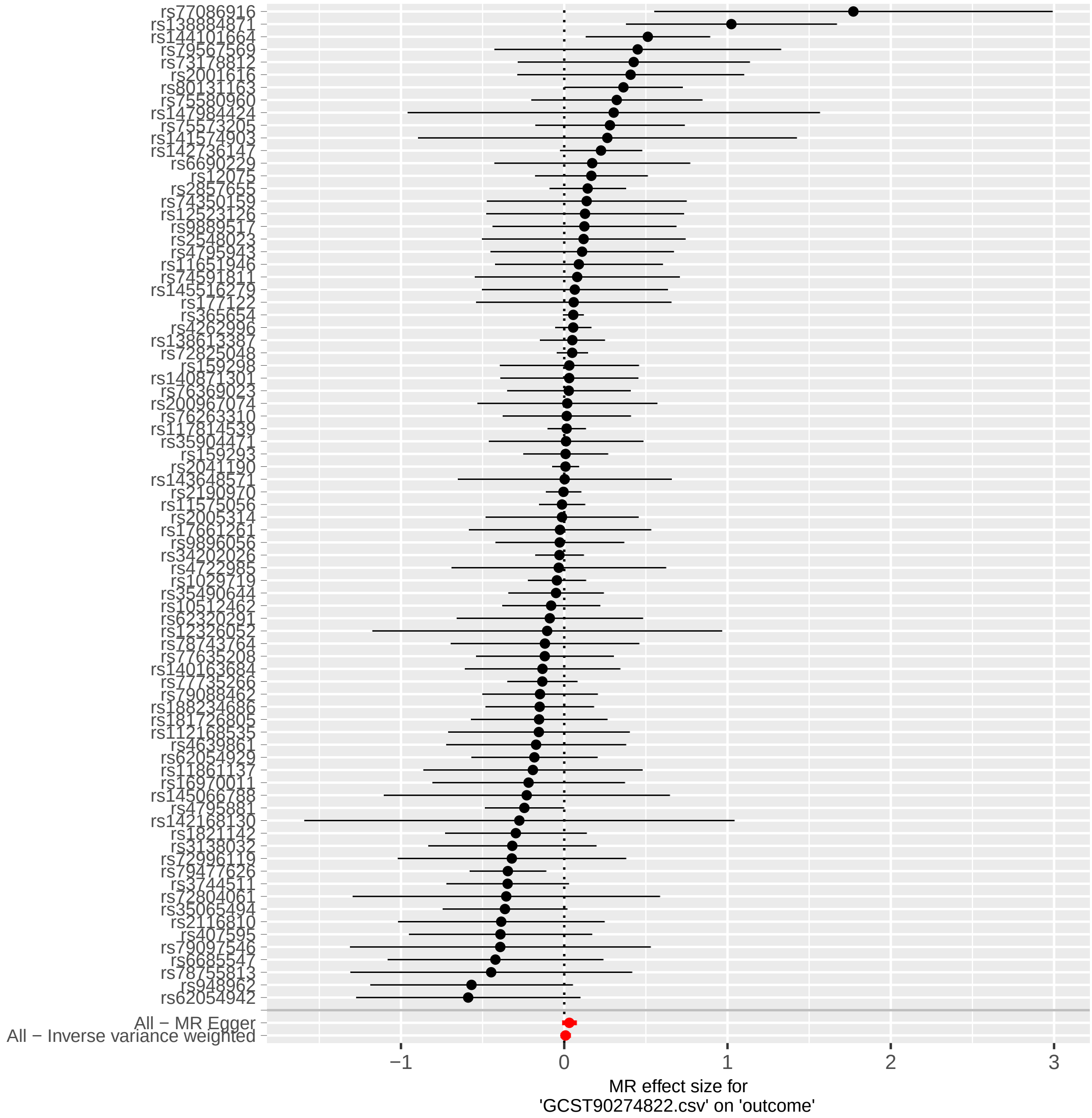
rs77086916
rs138884871
rs144101664
rs79567569
rs73178812
rs2001616
rs80131163
rs75580960
rs147984424
rs75573205
rs141574903
rs142736147
rs6690229
rs12075
rs2857655
rs74350159
rs12523126
rs9889517
rs2548023
rs4795943
rs11651946
rs74591811
rs145516279
rs177122
rs365654
rs4262996
rs138613387
rs72825048
rs159298
rs140871301
rs76369023
rs200967074
rs76263310
rs117814539
rs35904471
rs159293
rs2041190
rs143648571
rs2190970
rs11575056
rs2005314
rs17661261
rs9896056
rs34202026
rs4722985
rs1029719
rs35490644
rs10512462
rs62320291
rs12326052
rs78743764
rs77635208
rs140163684
rs77735266
rs79088462
rs188234686
rs181726805
rs112168535
rs4639861
rs62054929
rs11861137
rs16970011
rs145066788
rs4795881
rs142168130
rs1821142
rs3138032
rs72996119
rs79477626
rs3744511
rs72804061
rs35065494
rs2116810
rs407595
rs79097546
rs6685547
rs78755813
rs948962
rs62054942
All − MR Egger
All − Inverse variance weighted
−1
0
1
2
3
MR effect size for
'GCST90274822.csv' on 'outcome'
